# The Medical Library Association Guide to Developing Consumer Health Collections

**DOI:** 10.5195/jmla.2019.638

**Published:** 2019-04-01

**Authors:** Martha F. Earl

**Affiliations:** Preston Medical Library, Graduate School of Medicine, University of Tennessee, Knoxville, TN, mearl@utmck.edu

Health sciences librarians have been providing consumer and patient health information services for decades. To provide that information, collection development remains imperative, particularly in the light of the increase in fake health news sources and increasing disconnect between science and the public. In her preface, Claire B. Joseph, AHIP, director of the Harbhajan Singh, MD Medical Library at South Nassau Communities Hospital, Oceanside on Long Island, defines “consumer health library” in broad terms, including any collection, either print or electronic, in any library type. She aims with this book to provide an information resource for all librarians, though her focus is on the novice. She defines the two major challenges for libraries as the pervasive use of “Dr. Google” by all segments of society and the lack of access to health care and information for the medically underserved. Partnerships and outreach, with examples provided by the author, can help to overcome those challenges.

As a reference work, each chapter of the book serves as a tool and begins with italicized take-away points. References from each chapter include current articles, websites, and texts. Joseph writes in a clear, well-organized style, using practical tips and copious examples to illustrate concepts. She presents policies, models, and forms along with illustrations that increase the readability of the already engaging text. She provides lists of figures, tables, and sample forms at the beginning of the book. Even seasoned consumer health librarians can come away with new knowledge and ideas.

Eleven chapters expertly cover the range of topics in consumer health information. Chapter 1 details the main ingredients needed to begin, including assessing community needs, establishing relationships with stakeholders, writing a strategic plan, developing a budget, and planning space. Chapter 2 delves into how different types of communities impact health and how librarians can use that knowledge to tailor collections and services. Chapter 3 explores online and print sources and creation of mission statements and collection development policies. Chapter 4 covers grant writing. Chapter 5 focuses on staff development for customer service with specific training needs detailed for all types of library staff, from volunteers to professionals. Chapter 6 addresses library privacy and confidentiality. Chapter 7 provides step-by-step instructions for community outreach planning, including how to write a logic model, measure results, and manage market outreach. Chapter 8 covers health literacy, from definitions to impact on populations and individuals, and how librarians can work with consumers, institutions, and health professionals to address this key issue. Chapter 9 investigates resources for finding multicultural and inclusive consumer health information, including foreign language materials, but stresses that staff must create an inviting and accepting environment for diverse peoples along with materials to meet their information needs. Chapter 10 addresses mobile apps, Wikipedia, and social media with valuable insight on how to steer consumers to more valid sources and how to use social media to promote the library. Chapter 11 describes consumer health information outreach programs for libraries of any size or budget with intriguing examples of how libraries have successfully provided outreach.

Joseph believes that real-life examples constitute one of the best ways to learn. She undergirds each chapter with relevant and creative examples. A librarian for over forty years, Joseph skillfully focuses on what libraries are doing now and how any library type can learn from the innovative ideas of others. She also provides an index that includes key authors cited, as well as major topics.

This publication represents the new standard in creating or revitalizing consumer health collections and services. As one of the guides chosen by the Medical Library Association Books Panel, this text can be employed by library and information science educators to train future consumer health librarians and by practicing librarians in any library type.

This book is highly recommended for any health sciences, academic, public, school, or special library developing any consumer health collection, service, or outreach. University libraries supporting schools of information science, public health, or health education may also find it valuable.

